# U-shaped relationship between serum uric acid and gastric cancer risk: a large prospective cohort study

**DOI:** 10.3389/fonc.2024.1482814

**Published:** 2024-12-23

**Authors:** Junjun Huang, Ningning Mi, Jingli Yang, Ya Zheng, Jinqiu Yuan, Wenbo Meng

**Affiliations:** ^1^ Scientific research & Planning Department, The First Hospital of Lanzhou University, Lanzhou, China; ^2^ The First Clinical Medical School, Lanzhou University, Lanzhou, China; ^3^ College of Earth and Environmental Sciences, Lanzhou University, Lanzhou, China; ^4^ Key Laboratory for Gastrointestinal Diseases of Gansu Province, The First Hospital of Lanzhou University, Lanzhou, China; ^5^ Clinical Research Center, The Seventh Affiliated Hospital, Sun Yat-sen University, Shenzhen, China; ^6^ Scientific Research Center, The Seventh Affiliated Hospital, Sun Yat-sen University, Shenzhen, China; ^7^ Department of General Surgery, The First Hospital of Lanzhou University, Lanzhou, China; ^8^ Institute of Genetics, School of Basic Medical Sciences, Lanzhou University, Lanzhou, China; ^9^ Gansu Province Institute of Hepatopancreatobiliary, Lanzhou, China; ^10^ Gansu Province Key Laboratory Biotherapy and Regenerative Medicine, Lanzhou, China

**Keywords:** upper gastrointestinal cancer, uric acid, UK biobank, cohort study, U-shaped relationship

## Abstract

**Objective:**

We conducted this study to investigate the relationship between serum uric acid (SUA) levels and the risk of upper gastrointestinal cancer.

**Methods:**

We conducted a prospective cohort study with 475659 cancer-free participants from the UK Biobank. All subjects were grouped into quartiles, and we used a Cox proportional hazards model to analyze the association between SUA levels and the risk of upper gastrointestinal cancer and explore the potential sex-specific relationship.

**Results:**

Of the 475659 participants, 883 eventually developed upper gastrointestinal cancers over a median follow-up period of 6.7 years. We observed that SUA level was positively correlated with the risk of female oral cancer (hazard ratio _Quartile 4 vs Quartile 1_ (95% CI): 2.05(1.03,4.06)) and negatively associated with the risk of esophageal cancer in the general population (hazard ratio _Quartile 3 vs Quartile 1_ (95% CI): 0.65(0.45,0.93)). The risk of gastric cancer in males showed a U-shaped trend, decreasing and then increasing as SUA levels increased (hazard ratio _Quartile 3 vs Quartile 1_ (95% CI): 0.51(0.32,0.81)). The risk of small intestine cancer in females showed a trend of increasing and then decreasing with increasing SUA levels (hazard ratio _Quartile 3 vs Quartile 1_ (95% CI): 3.34(1.10,10.13)). Interaction analysis indicated that various factors, such as age, sex, smoking and drinking status, family history of cancer and BMI might play an important role in the relationship between SUA and cancer.

**Conclusion:**

SUA levels are positively associated with the risk of oral cancer risk in females and negatively associated with the risk of esophageal cancer in the general population. Both low and high SUA levels were associated with increased risk of gastric cancer, supporting a U-shaped association.

## Introduction

Cancer has always been one of the most significant threats to human health. According to the World Health Organization, digestive system cancer accounts for three of the top ten cancers in terms of incidence. In the male population, even half of the top ten cancers are digestive cancers in terms of incidence.

Uric acid, which is mainly present in the blood in the form of urate, is an end product of purine metabolism through the liver and is excreted by the kidneys and gut. The dominant source of uric acid (about two-thirds or more) is generated from endogenous purines and the rest from the exogenous (1, 2). Several studies have found that SUA is associated with the development of many human diseases (3, 4). Since SUA is an antioxidant, it should theoretically have anti-tumor effects (5). However, researches on the relationship between SUA and cancer risk indicates that SUA not only plays an antioxidant and anticancer role in the process of cancer occurrence (6). Emerging studies propose a new hypothesis that uric acid is also associated with oxidative stress in the body, which can lead to DNA damage, oxidation, production of inflammatory cytokines, and even cell apoptosis (7). SUA is a marker of chronic inflammation and therefore should be associated with an increased risk of cancer (8). Epidemiological studies have also found different results for the effects of SUA on cancer. A large European cohort study found that higher levels of uric acid are related to lower risks of breast cancer and cancer mortality (9). Another prospective cohort study from UK Biobank found that high SUA is associated with a high incidence of kidney cancer, especially in women (10). SUA appears to play different roles in different cancers and different populations, suggesting that the mechanism of SUA in cancer is very complicated. Therefore, more in-depth and detailed research is needed.

The UK Biobank is a prospective cohort study with deep genetic, physical and health data collected on more than 500,000 individuals across the United Kingdom (11). The database is regularly augmented with additional data and is globally accessible to approved researchers undertaking vital research into the most common and life-threatening diseases. Our research group has previously completed research on the relationship between SUA and the risk of liver cancer, gallbladder cancer, pancreatic cancer, rectal cancer and colon cancer depending on UK Biobank (12, 13). And this study aimed to investigate the relationship between SUA and the development of upper gastrointestinal cancer.

## Method

### Study population

This study used data from the UK Biobank, which is a large-scale biomedical database and research resource, containing in-depth genetic and health information from half a million participants aged 40-69 years. The participants were recruited throughout England, Wales, and Scotland between 2006 and 2010, which make sure that all participants were widely distributed in order to reliably detect the association between baseline characteristics and health outcomes. Each participant completed a touchscreen questionnaire, nurse-led interview and provided physical measures and biological samples at baseline assessment. The North West Multi-center Research Ethics Committee has approved the UK Biobank study, and all participants provided written informed consent before data collection (14, 15).

A total of 502527 participants were included in this study, with 26,868 excluded due to a pre-existing cancer diagnosis at baseline. To minimize follow-up time bias (16), 8,049 participants with less than two years of follow-up time were also excluded. Furthermore, 30643 participants with missing SUA data were excluded from the study. Ultimately, this study included a total of 436,964 subjects, including 231,595 males and 235,369 females.

### Covariates

Most of the covariates in this study were collected via participant interviews at baseline. These covariates included age (calculated based on the birth age provided by the participants), gender, alcohol intake (categorized as daily or almost daily, never, once or twice a week, one to three times a month, special occasions only, or three or four times a week), smoking status(categorized as current, previous, or never), Household income data (categorized as less than £18 000, £18 000 to £30 999, £31 000 to £51 999, £52 000 to £100 000, or greater than £100 000), ethnicity(categorized as white, Asian or Asian British, or black or black British), body mass index (BMI), fruit and vegetable intake (categorized as more than five portions per day or not), family history of cancer, meat intake(categorized as high, moderate, or low), education (categorized as A levels/AS levels or equivalent, College or University degree, CSEs or equivalent, NVQ or HND or HNC or equivalent, O levels/GCSEs or equivalent, or other professional qualifications).

SUA was measured using an enzymatic determination (Uricase PAP) on a Beckman Coulter AU5800 instrument (BC, USA). Measurement details can be found on the website of UK Biobank (www.ukbiobank.ac.uk).

### Outcomes

Information about cancer incidence in participants was obtained from the UK Biobank’s Health and Social Care Information Centre (in England and Wales) and the National Registry of Health Services (in Scotland). Because we did not have baseline data for jejunum, ileum and duodenum cancers, we included small intestine cancer in upper gastrointestinal cancer. Finally, oral cancer, esophageal cancer, gastric cancer and small intestine cancer were involved in our study. Cancers were coded by the 10^th^ revision of the international classification of Diseases (ICD-10) in these registries. The endpoints of this study were oral cancer (CD06), esophageal cancer (CD15), gastric cancer (CD16), and small intestine cancer (CD17).

### Statistical analyses

All participants in this study were divided into quartiles based on their SUA levels. Continuous variables are presented as means (standard deviation, SD), and categorical variables are represented as numbers (percentages). To examine the associations between SUA levels and the endpoints (oral, esophageal, gastric and small intestine cancer), Cox proportional hazards regression models were used and the results were presented with HRs and 95% CI. we used three models to adjust in sequence for potential confounding factors that could have influenced the results. In model 1, we adjusted for demographic characteristics (age, gender, education, ethnicity, and family history of cancer). Then we further adjusted for lifestyle factors (smoking status, alcohol consumption, fruit intake, annual household income and physical activity) in model 2. Because BMI is a significant confounding factor in the relationship between SUA and cancer risk, we separately adjusted BMI in model 3 based on model 2. Finally, in mode 3, we adjusted for age, gender, alcohol consumption, smoking status, education, ethnicity, family history of cancer, fruit intake, annual household income and physical activity. The potential linear relationships between the SUA levels and the cancer risk were investigated by fitting restricted cubic splines in a fully adjusted Cox regression model. In addition, we conducted an analysis based on gender stratification. R software (version 3.5.3, R Foundation for Statistical Computing, Vienna, Austria) was used for the data analysis. A two-sided P value of <0.05 was considered statistically significant.

## Results

A total of 436964 subjects were included in this study, comprising 201595 males and 235369 females. Among all participants, 883 eventually developed upper gastrointestinal cancers over a median follow-up period of 6.7 years. Of these, 209 participants were diagnosed with oral cancer, 363 with esophageal cancer, 234 with gastric cancer, and 77 with small intestine cancer.

Compared to the group with lower uric acid levels, participants in the group with higher SUA levels had a higher average age. In the group with higher uric acid levels, the proportion of male participants (80.7 percent in the fourth quartile) was significantly higher than that of female participants (19.3 percent in the fourth quartile). Those who had smoked before, drink daily or almost daily, consume less fruit, have family history of cancer, and those who exercise too much all accounted for a high proportion in the quartiles with higher SUA levels (The baseline characteristics are presented in **Table 1**).

**Table 1:** Baseline characteristics of total population.

Characteristics	Q1(N=109204)	Q2(N=109221)	Q3(N=109277)	Q4(N=109262)
Age				
Mean (SD)	55.2 (8.20)	56.8 (8.00)	57.4 (7.98)	57.6 (8.01)
Gender				
Female	97698 (89.5%)	72964 (66.8%)	43583 (39.9%)	21124 (19.3%)
Male	11506 (10.5%)	36257 (33.2%)	65694 (60.1%)	88138 (80.7%)
Drinking status				
Daily or almost daily	17072 (15.6%)	19887 (18.2%)	23234 (21.3%)	28451 (26.0%)
Smoking status				
Current	11354 (10.4%)	11530 (10.6%)	11874 (10.9%)	11230 (10.3%)
previous	31291 (28.7%)	34602 (31.7%)	38437 (35.2%)	44874 (41.1%)
Never	66076 (60.5%)	62550 (57.3%)	58382 (53.4%)	52577 (48.1%)
Income				
18,000 to 30,999	22870 (20.9%)	23823 (21.8%)	23851 (21.8%)	23681 (21.7%)
31,000 to 51,999	24326 (22.3%)	24291 (22.2%)	24851 (22.7%)	24856 (22.7%)
52,000 to 100,000	19387 (17.8%)	18571 (17.0%)	19339 (17.7%)	20319 (18.6%)
Greater than 100,000	5068 (4.6%)	4885 (4.5%)	5157 (4.7%)	5616 (5.1%)
Less than 18,000	20325 (18.6%)	21033 (19.3%)	20805 (19.0%)	21007 (19.2%)
Eth				
white	103168 (94.5%)	102716 (94.0%)	102570 (93.9%)	102648 (93.9%)
Asian or Asian British	1785 (1.6%)	2116 (1.9%)	2422 (2.2%)	2486 (2.3%)
Black or Black British	1655 (1.5%)	1841 (1.7%)	1760 (1.6%)	1709 (1.6%)
BMI				
Mean (SD)	25.1 (4.00)	26.8 (4.46)	28.1 (4.62)	29.6 (4.81)
Fruit & vegetable intake				
>=5 portions	46864 (42.9%)	43498 (39.8%)	39358 (36.0%)	34982 (32.0%)
<5 portion	62105 (56.9%)	65444 (59.9%)	69603 (63.7%)	73953 (67.7%)
Family history of cancer				
Yes	37340 (34.2%)	38195 (35.0%)	38675 (35.4%)	38270 (35.0%)
No	70178 (64.3%)	68974 (63.2%)	68161 (62.4%)	68079 (62.3%)
Physical activity				
High	35656 (32.7%)	36170 (33.1%)	36354 (33.3%)	35449 (32.4%)
Low	14546 (13.3%)	15709 (14.4%)	16814 (15.4%)	19113 (17.5%)
Educational level				
College or University degree	37873 (34.7%)	35978 (32.9%)	35345 (32.3%)	33351 (30.5%)

**Table 2** presents the results of the relationship between SUA level and cancer risk in the total population. We found that the incidence of upper gastrointestinal cancer was higher in the quartile with higher SUA levels. Cox regression analysis showed that compared to the first quartile, the risk of oral cancer was higher in the quartiles with higher SUA levels (P _linear in model 3_<0.001). In contrast, the risk of esophageal (hazard ratio _Quartile 3 vs Quartile 1_ (95% CI): 0.65(0.45,0.93)), and gastric cancer gradually decreased with increasing SUA levels (P _linear in model 3_<0.001). We did not find a significant relationship between SUA and the risk of small intestine cancer in the total population (hazard ratio _Quartile 4vs Quartile 1_ (95% CI): 1.32(0.58,3.04)). After adjusting for possible confounding factors, there was no significant change in the relationship between SUA levels and the risk of these four cancers.

**Table 2:** Effect of uric acid on upper gastrointestinal cancer in total population.

Cancer	Cases	Incidence*	HR (95%CI)	HR (95%CI)	*P* value
**Oral cancer**			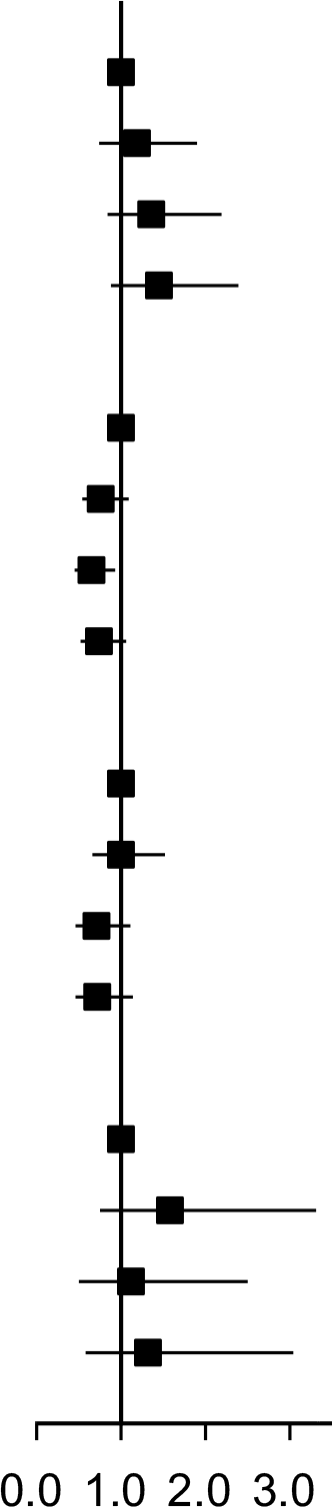		
Q1	31	4.261826		1.00	
Q2	45	6.221945		1.19 (0.74-1.90)	0.478
Q3	61	8.369453		1.36 (0.84-2.19)	0.207
Q4	72	9.976199		1.45 (0.88-2.39)	0.148
**Esophageal cancer**					
Q1	57	7.83626		1.00	
Q2	75	10.36991		0.76 (0.54-1.09)	0.139
Q3	92	12.62278		0.65 (0.45-0.93)	0.018
Q4	139	19.25961		0.74 (0.52-1.06)	0.101
**Gastric cancer**					
Q1	38	5.224173		1.00	
Q2	59	8.157661		1.00 (0.66-1.52)	0.999
Q3	60	8.232249		0.71 (0.46-1.11)	0.132
Q4	77	10.66899		0.72 (0.46-1.14)	0.160
**Small intestine cancer**					
Q1	12	1.649739		1.00	
Q2	22	3.04184		1.58 (0.75-3.31)	0.225
Q3	19	2.606879		1.12 (0.50-2.50)	0.789
Q4	24	3.3254		1.32 (0.58-3.04)	0.512
				

adjusted for age, sex, education, ethnic group and family history of cancer, alcohol intake, smoking status, annual household income, fruit and vegetable intake and physical activity, body mass index. *The incidence of cancer per 100000 person-years.

**Figure 1:**
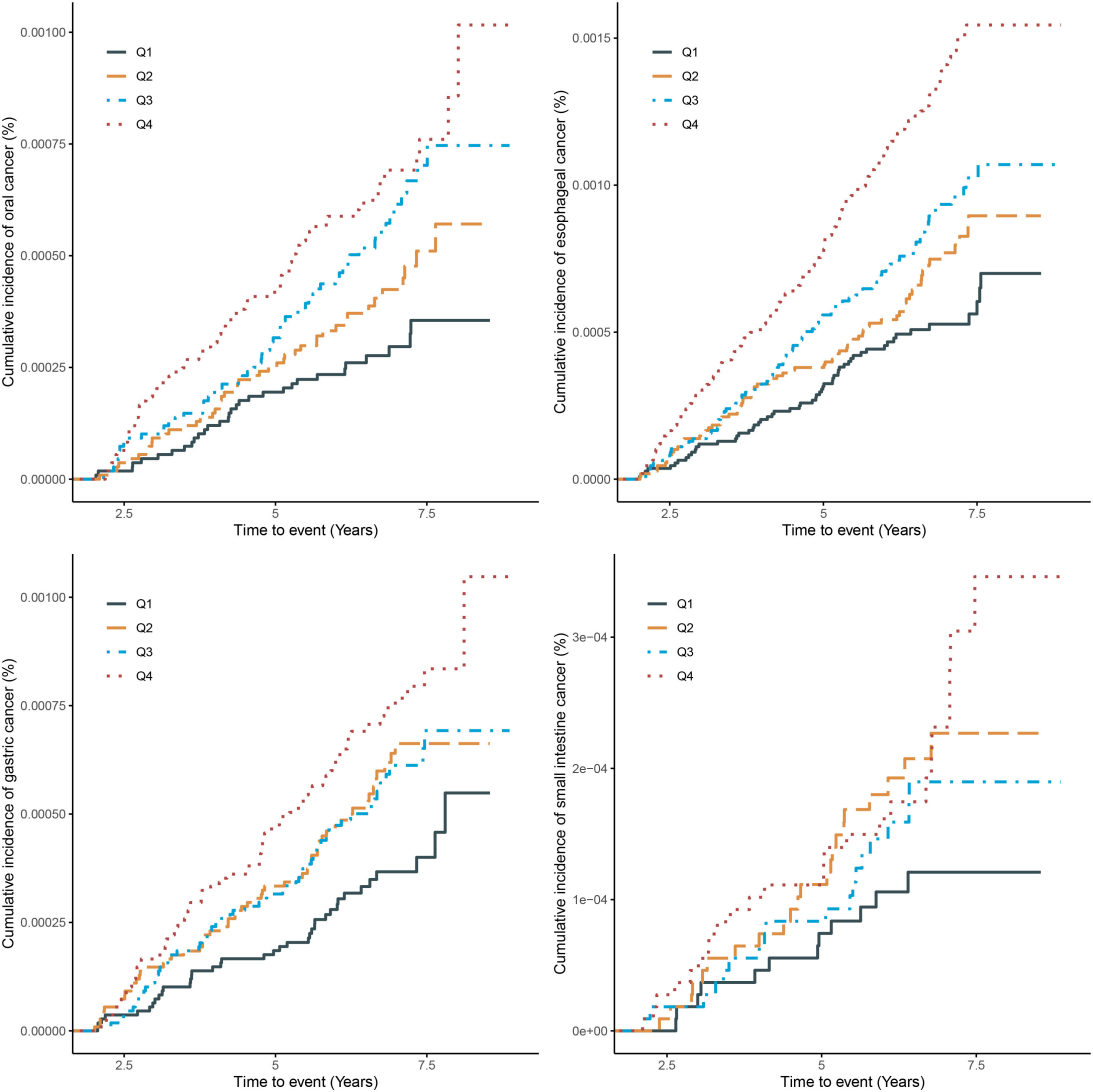
Kaplan-Meier curve for incidence of upper gastrointestinal cancer in total population.

The Kaplan-Meier curve (**Figure 1**) showed that the incidence of oral cancer, gastric cancer and esophageal cancer were higher in the quartile with higher SUA levels in the total population. However, the incidence of small intestine cancer in the second quartile is higher than in the third quartile.

We further conducted a gender stratified analysis, and present the results for the male population in **Table 3**. The findings showed that with increasing SUA levels, the incidence of small intestine cancer gradually increased, while the incidence of oral cancer, esophageal cancer and gastric cancer initially decreased and then increased in male participants. Cox regression analysis suggested that compared with the first quartile, the risk of male oral cancer, esophageal cancer and gastric cancer initially decreased and then increased with increasing SUA levels (**Figure 1**, **Supplementary Figure S1**) (P-overall<0.01, P-nonlinear<0.05). In the third quartile, the risk of these three cancers reached the lowest and the corresponding hazard ratios in model 3 were 0.79 (95% CI:0.48-1.32), 0.82 (95% CI:0.57-1.17) and 0.51 (95% CI:0.32-0.81). For male small intestine cancer, the corresponding hazard ratios gradually increased as SUA levels increased in model 1 and model 2. However, when we additionally adjusted for BMI in the third model, the trend was no longer evident (P _linear in model 3_ = 0.0898) and the hazard ratios for the fourth quartile decreased compared with the third quartile.

**Table 3:** Effect of uric acid on upper gastrointestinal cancer in males.

Cancer	No. of cases	Incidence (per 100000 person-years)	HR (95%CI)		
			model 1	model 2	model 3
Oral cancer (P _linear in model 3_<0.001)					
Q1	36	10.77428	1	1	1
Q2	35	10.53489	0.98 (0.61-1.56)	1.05 (0.66-1.67)	1.03 (0.64-1.64)
Q3	27	8.096325	0.75 (0.45-1.23)	0.82 (0.50-1.36)	0.79 (0.48-1.32)
Q4	39	11.64781	1.06 (0.68-1.67)	1.16 (0.73-1.84)	1.10 (0.68-1.78)
Esophageal cancer (P _linear in model 3_<0.001)					
Q1	67	20.05214	1	1	1
Q2	61	18.36081	0.91 (0.65-1.29)	0.96 (0.68-1.36)	0.91 (0.64-1.29)
Q3	60	17.99183	0.88 (0.62-1.25)	0.93 (0.66-1.32)	0.82 (0.57-1.17)
Q4	82	24.49026	1.16 (0.84-1.61)	1.22 (0.88-1.69)	1.01 (0.72-1.42)
Gastric cancer (P _overall in model 3_<0.01, P nonlinear<0.05)					
Q1	55	16.46071	1	1	1
Q2	36	10.83589	0.66 (0.43-1.00)	0.69 (0.45-1.05)	0.66 (0.43-1.00)
Q3	29	8.696053	0.52 (0.33-0.82)	0.56 (0.35-0.87)	0.51 (0.32-0.81)
Q4	44	13.14112	0.76 (0.51-1.13)	0.81 (0.54-1.21)	0.69 (0.45-1.05)
Small intestine cancer (P _linear in model 3_ = 0.0898)					
Q1	6	1.795714	1	1	1
Q2	8	2.407975	1.33 (0.46-3.82)	1.32 (0.46-3.80)	1.18 (0.41-3.40)
Q3	12	3.598367	1.94 (0.73-5.17)	1.91 (0.71-5.10)	1.52 (0.57-4.10)
Q4	13	3.882602	2.09 (0.79-5.49)	2.04 (0.77-5.42)	1.36 (0.50-3.71)

Model 1 adjusted for age, education, ethnic group and family history of cancer.

Model 2 adjusted for age, education, ethnic group, family history of cancer, alcohol intake, smoking status, annual household income, fruit and vegetable intake and physical activity.

Model 3 additionally adjusted for body mass index based on model 2.

In the female population (**Table 4**), we observed that as the SUA level increased, the incidence of oral cancer gradually increased, whereas the incidence of esophageal cancer gradually decreased, the incidence of gastric cancer showed a trend of first decreasing and then increasing, while the incidence of small intestine cancer showed a trend of first increasing and then decreasing. When compared with the first quartile with the lowest SUA level, the risk of oral cancer first decreased in the second quartile and then gradually increased in quartile 3 and quartile 4 (**Supplementary Figure S1**) (P _linear in model 3_ = 0.0038, hazard ratio _Quartile 4 vs Quartile 1_ (95% CI) = 2.05(1.03,4.06)). However, the risk of female small intestine cancer showed a trend of first increasing and then decreasing with the increase of SUA (**Supplementary Figure S1**) (the hazard ratio _Quartile 3 vs Quartile 1_ (95% CI) in model 3 is 3.34(1.10,10.13)). Although no significant results were found for esophageal cancer, its corresponding hazard ratios gradually decreased with increasing SUA levels (**Supplementary Figure S1**) (P _linear in model 3_ = 0.006, the hazard ratio _Quartile 4 vs Quartile 1_ (95% CI) in model 3 is 0.54(0.29,1.01)).

**Table 4:** Effect of uric acid on upper gastrointestinal cancer in females.

Cancer	No. of cases	Incidence (per 100000 person-years)	HR (95%CI)		
			model 1	model 2	model 3
Oral cancer (P _linear in model 3_ = 0.0038)					
Q1	15	3.803723	1	1	1
Q2	12	3.063555	0.76 (0.36-1.63)	0.77 (0.36-1.64)	0.80 (0.37-1.72)
Q3	15	3.827357	0.91(0.444-1.87)	0.92 (0.45-1.89)	1.00 (0.48-2.09)
Q4	30	7.720903	1.71 (0.91-3.22)	1.73 (0.92-3.26)	2.05 (1.03-4.06)
Esophageal cancer (P _linear in model 3_ = 0.006)					
Q1	26	6.593121	1	1	1
Q2	24	6.12711	0.82 (0.47-1.43)	0.82 (0.47-1.43)	0.80 (0.46-1.40)
Q3	21	5.3583	0.66 (0.37-1.17)	0.65 (0.36-1.16)	0.62 (0.35-1.13)
Q4	22	5.661995	0.60 (0.34-1.07)	0.58 (0.33-1.04)	0.54 (0.29-1.01)
Gastric cancer (P _linear in model 3_ = 0.0850)					
Q1	17	4.310887	1	1	1
Q2	10	2.552963	0.61 (0.28-1.32)	0.61 (0.28-1.33)	0.61 (0.28-1.32)
Q3	19	4.847986	0.96 (0.49-1.88)	0.97 (0.50-1.89)	0.96 (0.48-1.89)
Q4	24	6.176722	1.07 (0.56-2.03)	1.05 (0.55-2.00)	1.02 (0.51-2.05)
Small intestine cancer (P _linear in model 3_ = 0.9273)					
Q1	5	1.267908	1	1	1
Q2	8	2.04237	2.04 (0.63-6.62)	2.02 (0.62-6.56)	1.75 (0.52-5.83)
Q3	17	4.337671	3.59(1.20-10.71)	3.53(1.18-10.54)	3.34 (1.10-10.13)
Q4	8	2.058907	1.54 (0.46-5.15)	1.52 (0.45-5.09)	1.36 (0.38-4.81)

Model 1 adjusted for age, education, ethnic group and family history of cancer.

Model 2 adjusted for age, education, ethnic group, family history of cancer, alcohol intake, smoking status, annual household income, fruit and vegetable intake and physical activity.

Model 3 additionally adjusted for body mass index based on model 2.

**Figure 2:**
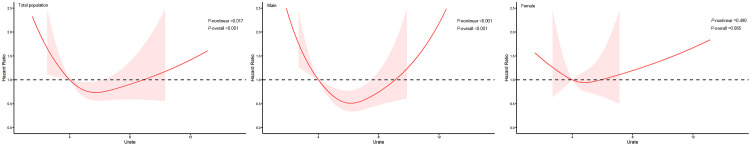
Dose response between serum uric acid and gastric cancer stratified by gender.

U-shaped association between serum uric acid and the risk of gastric cancer were found in general population and males (P-overall<0.01, P-nonlinear<0.05) after adjusted for age, education, ethnic group, family history of cancer, alcohol intake, smoking status, annual household income, fruit and vegetable intake, physical activity and BMI (**Figure 2**).

**Figure 3:**
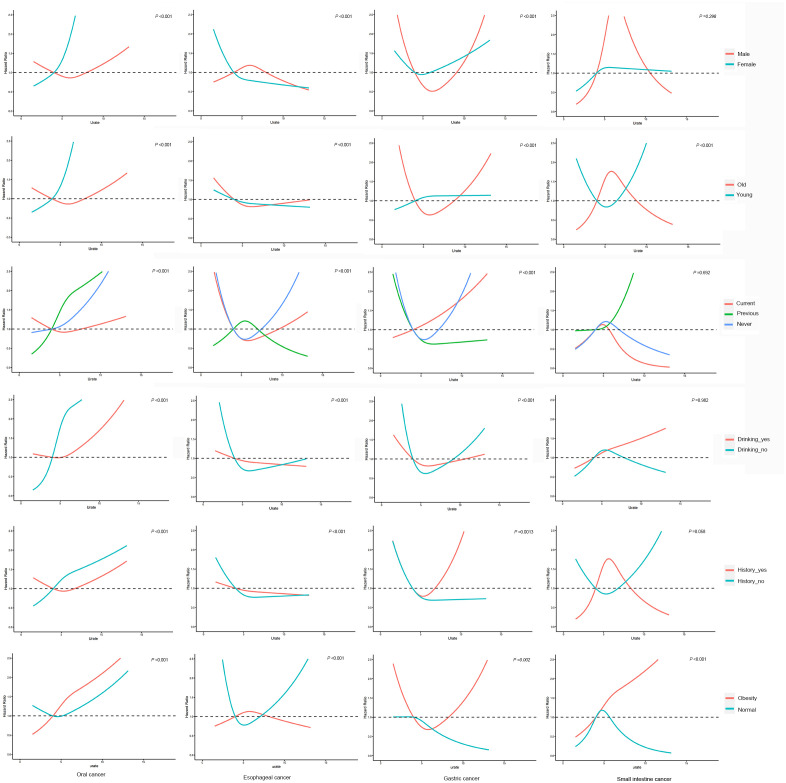
Interaction analysis. adjusted for age, education, ethnic group, family history of cancer, alcohol intake, smoking status, annual household income, fruit and vegetable intake, physical activity and BMI.

The results of the interaction analysis, as depicted in **Figure 3**, indicate that age and BMI interact with SUA in relation to the development of small intestine cancer, while gender, age, smoking and drinking status, family history of cancer and BMI all interact with SUA in relation to the development of oral, esophageal and gastric cancer (P _interaction_<0.001). Further investigation is needed to elucidate the underlying mechanisms of these interactions.

## Discussion

The results of this study showed that SUA levels were negatively associated with the risk of esophageal cancer. In the subgroup analysis by gender, we found that high SUA levels were a risk factor for the development of female oral cancer. Moreover, SUA levels showed a U-shaped association with gastric cancer in males, while the risk of female small intestine cancer showed a trend of first increasing and then decreasing with the increase of SUA levels.

To our knowledge, this study is the first to focus on the relationship between SUA and upper gastrointestinal cancer. Previous prospective cohort studies have found that high uric acid is associated with an increased risk of gout, kidney disease, cardiovascular disease and diabetes (3, 17, 18). However, studies on the relationship between SUA levels and cancer risk have yielded mixed results (6, 19, 20). For instance, a large prospective cohort study conducted based on UK Biobank found that high uric acid is associated with a high incidence of kidney cancer, especially in women (10). Andrew et al. found that elevated levels of SUA were associated with an increased cancer incidence, and gender stratification analysis showed that elevated SUA levels played a protective role in the risk of male central nervous system cancer, female breast cancer, melanoma, and central nervous system cancer (21). Our study showed that SUA has different effects on the risk of different kinds of cancers, and gender stratification analysis also showed that the effect of SUA on cancer varies between males and females. These findings suggest that the way SUA works on cancer is very complex and further research is necessary to reveal the mechanism.

SUA is traditionally considered to be a metabolically inert end-product of purine metabolism, without any physiological value (22). However, this ubiquitous compound has been proven to be a selective antioxidant, capable especially of reacting with hydroxyl radicals and hypochlorous acid. Due to this function, SUA was thought to reduce the risk of cancer caused by oxidation damages and radicals (5). On the other hand, elevated SUA levels have been found to be a marker of chronic inflammation, therefore it is suggested to be associated with an increased risk of cancer caused by chronic inflammation (8). Moreover, Rakesh et al. found a negative correlation between SUA levels and C-reactive protein levels in patients with head and neck tumors, indicating that SUA may also affect the risk of cancer by altering the levels of its risk factors (23). The research conducted by Liu et al. suggests that uric acid could be an important biomarker for cell death rather than an antioxidant for neural protection (24). In summary, uric acid may affect the risk of cancer through different pathways, with both promoting and anticancer effects present simultaneously. Therefore, the relationship between uric acid and the risk of cancers discovered in the present study should be the result of the co-action of multiple mechanisms of SUA.

In this study, SUA was found to have varying effects on different types of cancers and different populations, indicating that SUA may play different roles in different organs of the body, and the dominant mechanism of SUA on cancer varies in different organs. Interestingly, the risk of male gastric cancer and female small intestine cancer did not show a straightforward trend of increasing or decreasing with the increase of SUA levels. an investigation of 375,163 South Koreans showed that both low and high SUA levels were predictive of increased mortality, supporting a U-shaped association between serum uric acid levels and adverse health outcomes (25). Another cohort study conducted by Hu et al. on 9,118 US adults found similar results (26). This suggests that the effect of SUA on cancer may be closely related to its concentration, and the safe level of SUA is not unique, it’s not that lower is better, nor is it that higher is better.

The results of Interaction analysis in this study revealed that various factors, such as gender, age, smoking and drinking status, family history of cancer and BMI widely interact with SUA on the development of upper gastrointestinal cancer. This suggests that SUA not only affects the risk of cancer through its mechanism but also combines with other factors to produce a complex effect on the occurrence of cancer. Therefore, we should develop different SUA control plans based on each individual’s different characteristics to reduce the incidence rate of upper gastrointestinal cancer.

This is a large prospective cohort study with more than 500,000 individuals, such a large sample size allowed us to adequately control potential confounders and made the research results more credible. However, there are also limitations in this study. First, the present study is observational, and the results cannot establish a causal relationship between SUA and upper gastrointestinal cancer. Second, the participants of the UK Biobank were mainly white Europeans, and it is uncertain whether the results of this study can be applied to other ethnic groups. Third, the mechanism by which SUA affects upper gastrointestinal cancer was not clarified.

In conclusion, this study found a negative association between SUA levels and esophageal cancer, and a positive association between high SUA levels and female oral cancer; the risk of male gastric cancer and female small intestine cancer presented a non-linear trend with the increase of SUA levels. Our findings suggest that SUA levels may be a useful marker for predicting the risk of certain types of upper gastrointestinal cancer and that further basic experimental research is needed to understand the underlying mechanism of SUA on cancer development.

## Data Availability

The original contributions presented in the study are included in the article/**Supplementary Material**. Further inquiries can be directed to the corresponding authors.
