# Supplementary figures and images for: U-shaped relationship between serum uric acid and gastric cancer risk: a large prospective cohort study
Source: Front Oncol. 2024 Dec 23;14:1482814. doi: 10.3389/fonc.2024.1482814 (PMC11700790; doi:10.3389/fonc.2024.1482814)

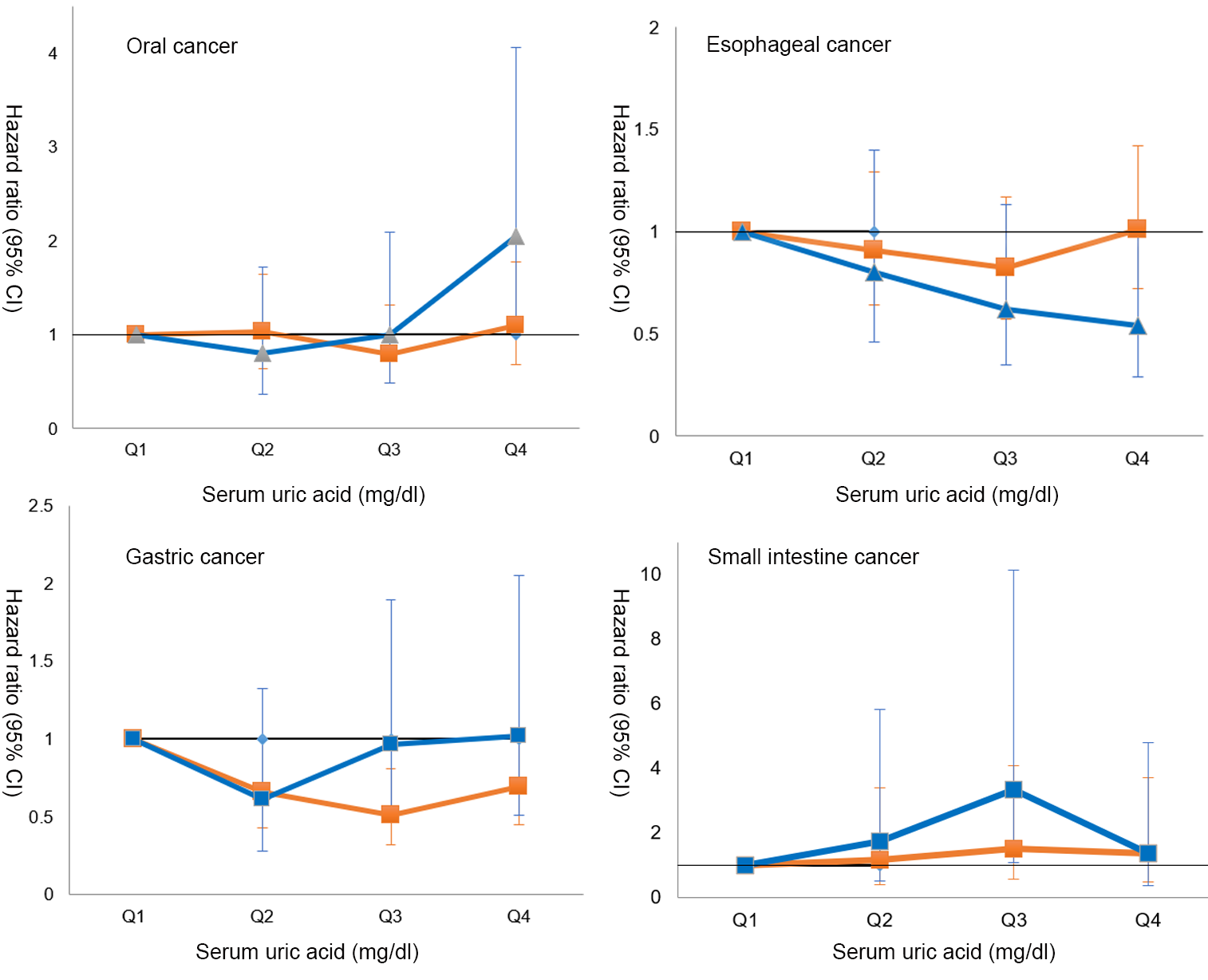

Supplement: Supplementary file 1 [file Image1.tif]
